# Directional couplings between the respiration and parasympathetic control of the heart rate during sleep and wakefulness in healthy subjects at different ages

**DOI:** 10.3389/fnetp.2022.942700

**Published:** 2022-09-06

**Authors:** Ekaterina I. Borovkova, Mikhail D. Prokhorov, Anton R. Kiselev, Aleksey N. Hramkov, Sergey A. Mironov, Mikhail V. Agaltsov, Vladimir I. Ponomarenko, Anatoly S. Karavaev, Oksana M. Drapkina, Thomas Penzel

**Affiliations:** ^1^ National Medical Research Center for Therapy and Preventive Medicine, Moscow, Russia; ^2^ Smart Sleep Laboratory, Saratov State University, Saratov, Russia; ^3^ Laboratory of Nonlinear Dynamics Modeling, Saratov Branch of Kotelnikov Institute of Radio Engineering and Electronics of Russian Academy of Sciences, Saratov, Russia; ^4^ Institute of Cardiological Research, Saratov State Medical University, Saratov, Russia; ^5^ Interdisciplinary Sleep Medicine Center, Charité—Universitätsmedizin Berlin, Berlin, Germany

**Keywords:** cardiovascular system, respiration, parasympathetic control of the heart rate, sleep studies, directional couplings

## Abstract

Cardiorespiratory interactions are important, both for understanding the fundamental processes of functioning of the human body and for development of methods for diagnostics of various pathologies. The properties of cardiorespiratory interaction are determined by the processes of autonomic control of blood circulation, which are modulated by the higher nervous activity. We study the directional couplings between the respiration and the process of parasympathetic control of the heart rate in the awake state and different stages of sleep in 96 healthy subjects from different age groups. The detection of directional couplings is carried out using the method of phase dynamics modeling applied to experimental RR-intervals and the signal of respiration. We reveal the presence of bidirectional couplings between the studied processes in all age groups. Our results show that the coupling from respiration to the process of parasympathetic control of the heart rate is stronger than the coupling in the opposite direction. The difference in the strength of bidirectional couplings between the considered processes is most pronounced in deep sleep.

## Introduction

The study of interaction between the human cardiac and respiratory systems attracts a lot of attention. The most studied types of cardiorespiratory interaction are the respiratory sinus arrhythmia (Angelone and Coulter, 1964; Schäfer et al., 1998; Song and Lehrer, 2003), which explains the variation of the heart rate within a breathing cycle, and the cardiorespiratory phase synchronization (Rosenblum et al., 1998; Schäfer et al., 1998; Schäfer et al., 1999; Mrowka et al., 2000; Prokhorov et al., 2003), which is defined as the occurrence of heartbeats in certain phases of the respiratory cycles. The methods based on calculation of cross-spectral coherence (White and Boashash, 1990; Quian Quiroga et al., 2002) and detection of synchronization (Rosenblum et al., 1998; Schäfer et al., 1999; Mrowka et al., 2000; Pikovsky et al., 2001; Rosenblum et al., 2001; Rosenblum and Pikovsky, 2001; Schelter et al., 2006a; Karavaev et al., 2009) helped to understand the cardiorespiratory interaction from a physiological point of view (Ivanov et al., 1998; Kantelhardt et al., 2002; Keener and Sneyd, 2009; Schumann et al., 2010). It has been shown that characteristics of the cardiorespiratory interaction change during sleep (Bunde et al., 2000; Kantelhardt et al., 2003; Bartsch et al., 2007; Schmitt et al., 2009; Schumann et al., 2010; Müller et al., 2014; Riedl et al., 2014; Karavaev et al., 2021) and during healthy aging (Bartsch et al., 2012; Ponomarenko et al., 2021), differ in newborns (Mrowka et al., 2000), and depend on the gender of the subjects (Shiogai et al., 2010). They can be used for predicting complications of cardiovascular diseases (Dougherty and Burr, 1992; Hohnloser et al., 1994; Ishbulatov et al., 2020) and help to understand the mechanism of neural control of the cardiovascular and respiratory systems (Sayers, 1973; Lown and Verrier, 1976; Akselrod et al., 1981; Task Force of the European Society of Cardiology and the North American Society of Pacing and Electrophysiology, 1996; Malberg et al., 2002; Prokhorov et al., 2005a; Karavaev et al., 2013; Ishbulatov et al., 2020).

In order to get a more detailed understanding of the mechanism of cardiorespiratory coupling, many authors study the driver-response (causal) relationships, or directionality of coupling using the methods based on Granger causality (Baccala et al., 1998; Baccala and Sameshima, 2001; Kaminski et al., 2001; Porta et al., 2002; Faes et al., 2004; Astolfi et al., 2006; Schelter et al., 2006b; Faes et al., 2006; Baccala et al., 2007; Faes and Nollo, 2010; Milde et al., 2011; Porta et al., 2012; Schulz et al., 2013; Faes et al., 2015), entropy (Schreiber, 2000; Hoyer et al., 2002; Kaiser and Schreiber, 2002; Bhattacharya et al., 2003; Ancona et al., 2004; Verdes, 2005; Bahraminasab et al., 2008; Faes et al., 2011), and modeling of phase dynamics (Schäfer et al., 1998; Censi et al., 2002; Rosenblum et al., 2002; Bartsch et al., 2007). It has been shown that in healthy infants, the direction of coupling between cardiovascular and respiratory systems evolves from approximately symmetric coupling during the first days of life to nearly unidirectional (from respiration to the cardiovascular system) after 6 months of age (Rosenblum et al., 2002). For a large database of healthy subjects, it has been shown that the intensity of influence is much stronger from respiration to heart than in the opposite direction and the direction of coupling from respiration to the main heart rhythm is dominant throughout life (Faes et al., 2004; Faes and Nollo, 2010; Porta et al., 2012) and does not depend on the subject’s gender (Mrowka et al., 2003; Bartsch et al., 2007; Bahraminasab et al., 2008; Shiogai et al., 2010) or sleep stage (Mrowka et al., 2003). However, the intensity of influence from respiration to heart decreases with age (Shiogai et al., 2010) and during active standing or head-up tilt protocols (Nollo et al., 2005; Faes et al., 2011; Porta et al., 2012; Faes et al., 2015) and changes under anesthesia (Stankovski et al., 2016). On the other hand, the intensity of influence from heart to respiration remains constant with age (Iatsenko et al., 2013). In spontaneously breathing patients under general anesthesia (Galletly and Larsen, 1999) and in the case of so-called dynamic diseases such as apnea, the mechanisms of cardiorespiratory interaction and feedback between heart rate and respiration are disrupted, leading to an increase in the directional coupling from the main heart rhythm to respiration (Schreiber, 2000; Kaiser and Schreiber, 2002; Bhattacharya et al., 2003; Ancona et al., 2004; Verdes, 2005).

Causal relationships between the human cardiac and respiratory systems were studied mainly between the main heart rhythm with a frequency of about 1 Hz and respiration whose frequency is usually around 0.25 Hz. Another aspect of the cardiorespiratory interaction is associated with the relationships between the respiration and fluctuations of the heart rate in the high-frequency (HF) range 0.15–0.4 Hz. The occurrence of fluctuations in the sequence of RR-intervals in the HF range is associated with a number of factors, including the parasympathetic control of the heart rate (Task Force of the European Society of Cardiology and the North American Society of Pacing and Electrophysiology, 1996; Porges, 1995; Lewis et al., 2006; Prokhorov et al., 2021), intrathoracic pressure changes, and indirect influence of interaction between central generators of cardiorespiratory rhythms and peripheral factors (tonic and phasic baroreceptor and chemoreceptor reflexes, cardiac and pulmonary stretch reflexes, local chemical and metabolic factors, etc.) (Berntson et al., 1993). Recently, we have shown the decrease of coherence between the respiration and parasympathetic control of the heart rate with aging in healthy subjects (Ponomarenko et al., 2021). The coherence between these processes depends on the stage of sleep (Ponomarenko et al., 2021).

In this paper, we study the directional couplings between the respiration and the process of parasympathetic control of the heart rate in healthy subjects. We investigate whether these directional couplings depend on age and the stages of sleep.

## Materials and methods

### Study participants

Our study included 96 healthy subjects (59 females and 37 males), who were divided into four groups depending on age. The first group included 36 subjects aged 20–34 years, the second group included 23 subjects aged 35–49 years, the third group included 17 subjects aged 50–64 years, and the fourth group included 20 subjects in ages 65 and older. The data were recorded at the sleep laboratories within the European Union project SIESTA (Klösch et al., 2001). The study was approved by the local institutional review boards of the sleep centers involved. All study participants provided written informed consent. Exclusion criteria subjects for the healthy group were obstructive apnea and hypopnea and identified pathologies of the respiratory, cardiovascular, and neural system.

### Data preprocessing

The signals of respiration and electrocardiogram (ECG) were simultaneously recorded within 8 h at night for each subject. The respiratory signal was recorded with a sampling frequency of 20 Hz using a thermistor oronasal respiration flow sensor. The ECG signal was recorded with a sampling frequency of 200 Hz. We detected the epochs of wakefulness, rapid eye movement (REM) sleep, light sleep S2 (LS), and deep sleep S3 (DS) in accordance with the classification (Rechtschaffen and Kales, 1968). We analyzed the first 5-min segments of the detected epochs without artifacts in ECG and respiratory signals.

From the ECG signal, we extracted a sequence of RR-intervals, i.e., a series of time intervals between the two successive R peaks, in accordance with the standards of heart rate variability (HRV) measurement (Task Force of the European Society of Cardiology the North American Society of Pacing Electrophysiology, 1996). To obtain equidistant time series from not equidistant sequence of RR-intervals we approximated it with cubic splines and resampled with a frequency of 20 Hz.

To extract the high-frequency (HF) component of HRV associated with the process of parasympathetic control of the heart rate, we filtered the sequence of RR-intervals using a rectangular digital filter with the bandpass of 0.15–0.50 Hz. In a similar way, we filtered the respiratory signal with the same bandpass filter. The filtered signals of respiration and RR-intervals are denoted as *x*_1_(*t*) and *x*_2_(*t*), respectively.

### Indices of directional coupling

Using the filtered signals *x*_1_(*t*) and *x*_2_(*t*) we calculated the indices of directional coupling between the respiration and parasympathetic control of the heart rate during sleep and wakefulness in each subject. To calculate these indices, we used the method based on modeling the phase dynamics (Rosenblum and Pikovsky, 2001; Rosenblum et al., 2002; Smirnov and Bezruchko, 2003). The main idea of this method is to estimate how strongly the future evolution of the phase of the first (second) system depends on the current value of the phase of the second (first) system.

First, from time series of the signals *x*_1_(*t*) and *x*_2_(*t*), we obtain the time series of their instantaneous phases *φ*_1_(*t*) and *φ*_2_(*t*), respectively, using the Hilbert transform (Gabor, 1946; Panter, 1965; Pikovsky et al., 1997). Then, we construct stochastic differential equations modeling the phase dynamics of oscillatory processes:

$$d\varphi_{1,2}\left(t\right)/dt=\omega_{1,2}+G_{1,2}\left(\varphi_{1,2}\left(t\right),\varphi_{2,1}\left(t-\Delta \right)\right)+\xi_{1,2}\left(t\right),$$ (1)

where $\omega_{1,2}$ are the angular frequencies of oscillations, $G_{1,2}$ are 2π-periodic in both argument functions, Δ is the time shift between the time series of *φ*_1_(*t*) and *φ*_2_(*t*), and $\xi_{1,2}\left(t\right)$ are Gaussian white noises with zero mean (Smirnov et al., 2011; Sidak et al., 2017). Increments of phases during some fixed time interval *τ* can be estimated from experimental time series as follows:

$$\varphi_{1,2}\left(t+\tau \right)-\varphi_{1,2}\left(t\right)=F_{1,2}\left(\varphi_{1,2}\left(t\right),\varphi_{2,1}\left(t-\Delta \right),a_{1,2}\right)+\varepsilon_{1,2}\left(t\right),$$ (2)

where $F_{1,2}$ are the third-order trigonometric polynomials in the form proposed by (Rosenblum and Pikovsky, 2001), $a_{1,2}$ are vectors of their coefficients, and $\varepsilon_{1,2}\left(t\right)$ are Gaussian white noises with zero mean. Eq. 2 characterizes the dependence of phase increments (over a time interval *τ*) on the phases of systems’ oscillations. The time interval *τ* is taken to be equal to one characteristic period of oscillations (Rosenblum et al., 2002). The coefficients $a_{1,2}$ of the model equations are estimated from the time series of instantaneous phases using the least square method.

The intensity of influence of the second system on the first one, $c_{1}^{2}\left(\Delta \right)$ , is determined by the steepness of the dependence of $F_{1}$ on *φ*_2_, i.e., $\partial F_{1}/\partial \varphi_{2}$ . Similarly, the intensity of influence of the first system on the second one, $c_{2}^{2}\left(\Delta \right)$ , is determined by $\partial F_{2}/\partial \varphi_{1}$ :

$${с}_{1,2}^{2}\left(\Delta \right)=\int_{0}^{2\pi }\int_{0}^{2\pi }{\left(\partial F_{1,2}\left(\varphi_{1,2}\left(t\right),\varphi_{2,1}\left(t-\Delta \right),a_{1,2}\right)/\partial \varphi_{2,1}\right)}^{2}d\varphi_{1}d\varphi_{2}.$$ (3)

The derivatives $\partial F_{1,2}/\partial \varphi_{2,1}$ and the coupling indices ${с}_{1,2}^{2}\left(\Delta \right)$ depend on the variances $\sigma_{1,2}^{2}$ of the phases *φ*_1_(*t*) and *φ*_2_(*t*) and may differ even if there is no change in the couplings. We normalized the indices $c_{1}^{2}\left(\Delta \right)$ and $c_{2}^{2}\left(\Delta \right)$ to the variances $\sigma_{1}^{2}$ and $\sigma_{2}^{2}$ of instantaneous phases *φ*_1_(*t*) and *φ*_2_(*t*), respectively:

$$\rho_{1,2}\left(\Delta \right)=\frac{c_{1,2}^{2}\left(\Delta \right)}{\sigma_{1,2}^{2}}.$$ (4)

This normalization has disadvantages, since if the variances $\sigma_{1,2}^{2}$ change across comparisons then $\rho_{1,2}\left(\Delta \right)$ will also differ even if there is no change in the couplings. Although the normalized indices $\rho_{1,2}\left(\Delta \right)$ have the same disadvantage as the non-normalized indices ${с}_{1,2}^{2}\left(\Delta \right)$ , they allowed us to better distinguish between different stages of sleep and more clearly identify the asymmetry of couplings between the respiration and parasympathetic control of the heart rate than the indices ${с}_{1,2}^{2}\left(\Delta \right)$ .

The normalized index $\rho_{1}\left(\Delta \right)$ characterizes that part of variance of the respiratory signal phase, which can be described taking into account the phase of the signal of parasympathetic control of the heart rate shifted by time Δ with respect to the respiratory signal. The normalized index $\rho_{2}\left(\Delta \right)$ characterizes that part of variance of the phase of the signal of parasympathetic control of the heart rate, which can be described taking into account the phase of the respiratory signal shifted by time Δ. Thus, the indices $\rho_{1}\left(\Delta \right)$ and $\rho_{2}\left(\Delta \right)$ take into account the possible delay in coupling between the signals.

For each subject in the awake state and different stages of sleep, we varied the trial delay time Δ from −5 to 5 s and calculated the indices $\rho_{1}\left(\Delta \right)$ and $\rho_{2}\left(\Delta \right)$ . Then, we denote the maximal values of these indices as $\rho_{1\max}=\max\left(\rho_{1}\left(\Delta \right)\right)$ and $\rho_{2\max}=\max\left(\rho_{2}\left(\Delta \right)\right)$ , respectively. The positive values of both $\rho_{1\max}$ and $\rho_{2\max}$ indicate the presence of a bidirectional coupling between the respiration and the process of parasympathetic control of the heart rate. Close positive values of $\rho_{1\max}$ and $\rho_{2\max}$ correspond to symmetric coupling between the considered processes, while statistically significant different values of $\rho_{1\max}$ and $\rho_{2\max}$ indicate the presence of asymmetry in coupling. In this case, one of the directions of coupling is dominant. The case of only one positive index $\left(\rho_{1\max}\;\mathrm{or}\;\rho_{2\max}\right)$ corresponds to the unidirectional coupling. To compare $\rho_{1\max}$ and $\rho_{2\max}$ , we calculated the index *δ* characterizing their difference: $\delta =\rho_{2\max}-\rho_{1\max}$ .

### Statistical significance of estimated indices

To estimate a statistical significance of indices $\rho_{1\max}$ and $\rho_{2\max}$ calculated from experimental time series, we used surrogate data (Schreiber and Schmitz, 2000). We generated surrogate time series by random choice of signals *x*_1_(*t*) from one subject and signals *x*_2_(*t*) from another subject. For each state, we generated M=L(L−1)=9120 pairs of surrogates, where *L* = 96 is the number of subjects. Then, we calculated the indices $\rho_{1\max}^{i}$ and $\rho_{2\max}^{i}$ , i=1,…,M for each *i*th pair of surrogates. Over the whole ensemble of surrogates we plotted distributions of $\rho_{1\max}^{i}$ and $\rho_{2\max}^{i}$ values. A level *p* of statistical significance for the value of $\rho_{1\max}$ calculated from experimental data can be estimated as the ratio of area of distribution $P\left(\rho_{1\max}^{i}\right)$ corresponding to $\rho_{1\max}^{i}\geq \rho_{1\max}$ , to the entire area of distribution. Similarly, a *p*-value for the value of $\rho_{2\max}$ calculated from experimental data was computed. The indices $\rho_{1\max}$ and $\rho_{2\max}$ calculated from experimental time series were considered statistically significant if p≤0.05 , i.e., if $\rho_{1\max}$ and $\rho_{2\max}$ exceeded at least 95% of the indices $\rho_{1\max}^{i}$ and $\rho_{2\max}^{i}$ , respectively, calculated from surrogate data.

To evaluate a statistical significance of differences in the estimates of calculated indices in different groups of subjects, we used the Mann-Whitney U-test (Mann and Whitney, 1947).

## Results

**FIGURE 1:**
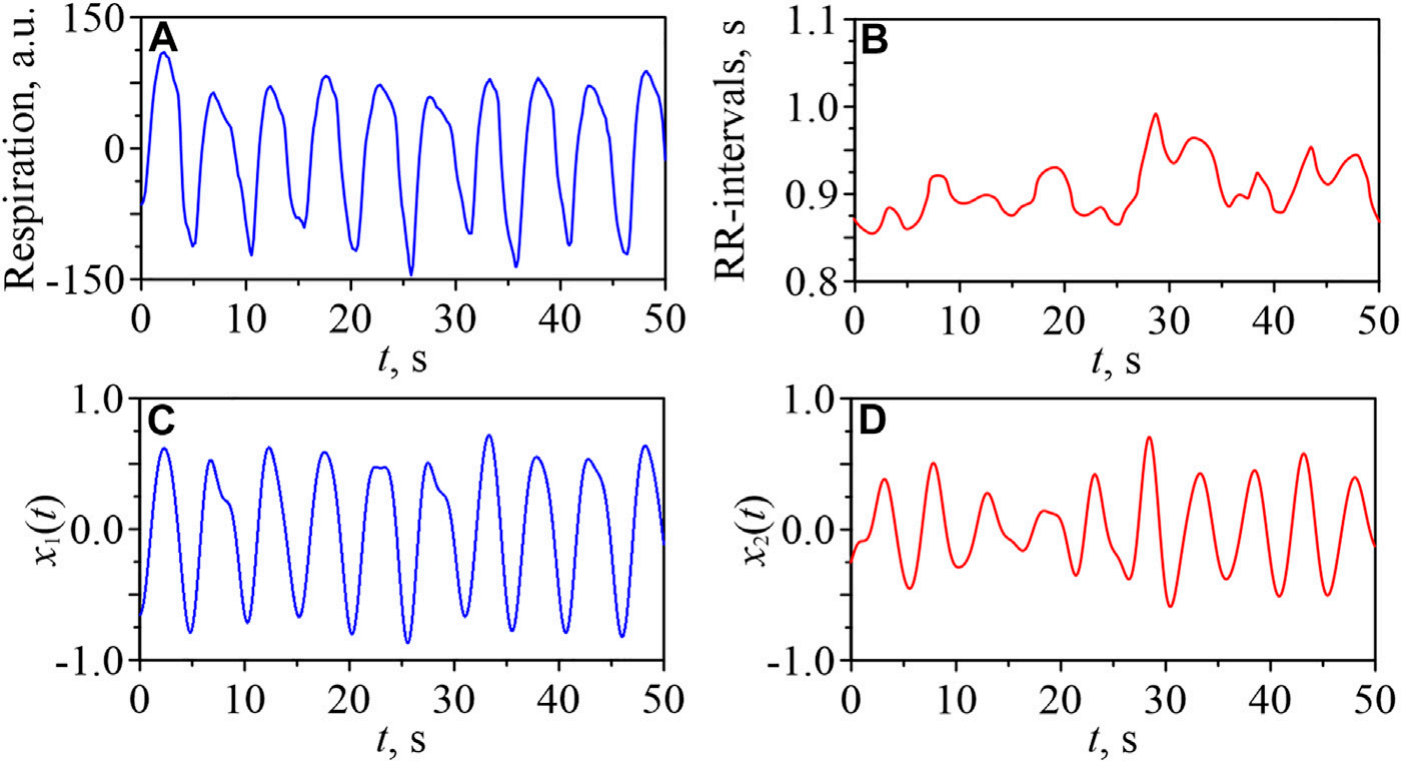
Fragments of a respiratory signal **(A)** and a sequence of RR-intervals **(B)** for one of the young subjects in the awake state. Filtered signals *x*_1_(*t*) **(C)** and *x*_2_(*t*) **(D)**.

Figure 1 shows short fragments of typical experimental signals for a healthy young subject in the awake state. The signal of respiration is presented in Figure 1A and the sequence of RR-intervals is presented in Figure 1B. Figures 1C,D show the time series of the signals *x*_1_(*t*) and *x*_2_(*t*), respectively, obtained by bandpass filtering of the signal of respiration and RR-intervals, respectively, in the 0.15–0.50 Hz band.

**FIGURE 2:**
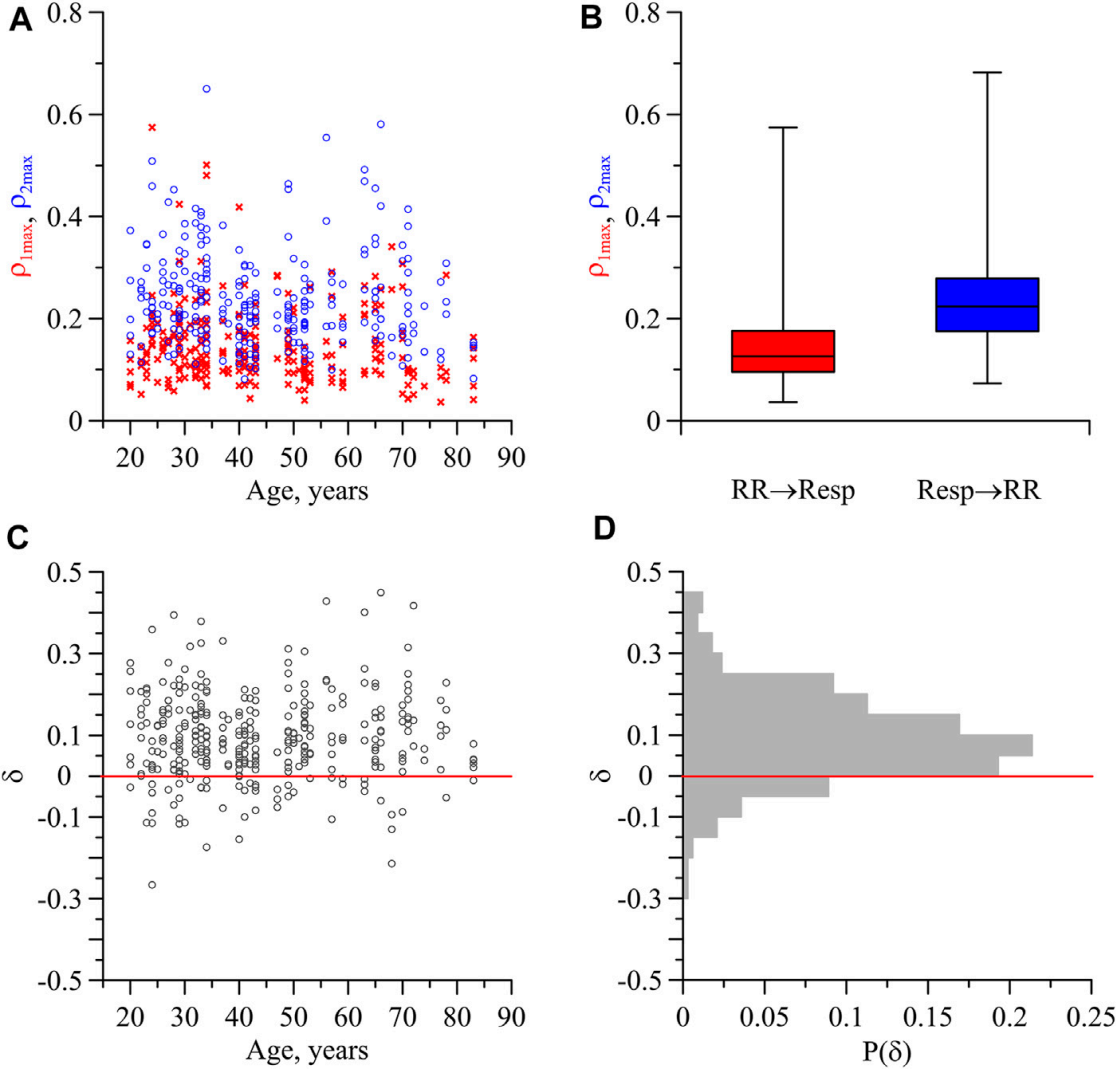
Indices of directional coupling between the respiration and the process of parasympathetic control of the heart rate. **(A)** Values of $\rho_{1\max}$ (red crosses) and $\rho_{2\max}$ (blue circles) for the subjects of different age. **(B)** Box-and-whisker diagrams for the values of $\rho_{1\max}$ and $\rho_{2\max}$ for all records. **(C)** Values of *δ* for the subjects of different age. **(D)** Distribution of index *δ*.

For each subject in the awake state, LS, DS, and REM sleep, we calculated the indices $\rho_{1\max}$ , $\rho_{2\max}$ , and *δ* for the signals *x*_1_(*t*) and *x*_2_(*t*) and estimated their statistical significance. The indices $\rho_{1\max}$ and $\rho_{2\max}$ turned out to be statistically significant (p≤0.05) that indicates the presence of a bidirectional coupling. Figure 2A presents only statistically significant (*p* = 0.05) values of $\rho_{1\max}$ and $\rho_{2\max}$ for all records of subjects from all age groups. The indices $\rho_{1\max}$ representing the direction of coupling from the HF-oscillations in RR-intervals to the respiration are shown by red crosses, while the indices $\rho_{2\max}$ representing the coupling in the opposite direction are shown by blue circles.

In Figure 2B, the box-and-whisker diagrams for the indices $\rho_{1\max}$ and $\rho_{2\max}$ are presented without taking into account the age of subjects and the stages of sleep or wakefulness. The box boundaries are the first and third quartiles, the horizontal line is the median, and the whiskers are the minimum and maximum values. In Figure 2B, the indices $\rho_{1\max}$ are lower (0.15 ± 0.01) than indices $\rho_{2\max}$ (0.24 ± 0.01). The measures are presented as mean ± standard error. The statistical significance of difference in indices $\rho_{1\max}$ and $\rho_{2\max}$ is confirmed by the Mann-Whitney U-test (*p*< 0.001).

Figure 2C shows the index *δ* characterizing the difference between the directional coupling indices $\rho_{2\max}$ and $\rho_{1\max}$ calculated for all records of subjects. Figure 2D depicts a distribution *P*(*δ*). It follows from Figure 2D that δ>0 in 84% of all analyzed records. Therefore, in these cases, the values of $\rho_{2\max}$ are greater than the values of $\rho_{1\max}$ , and the direction of coupling from respiration to the process of parasympathetic control of the heart rate is dominant.

**FIGURE 3:**
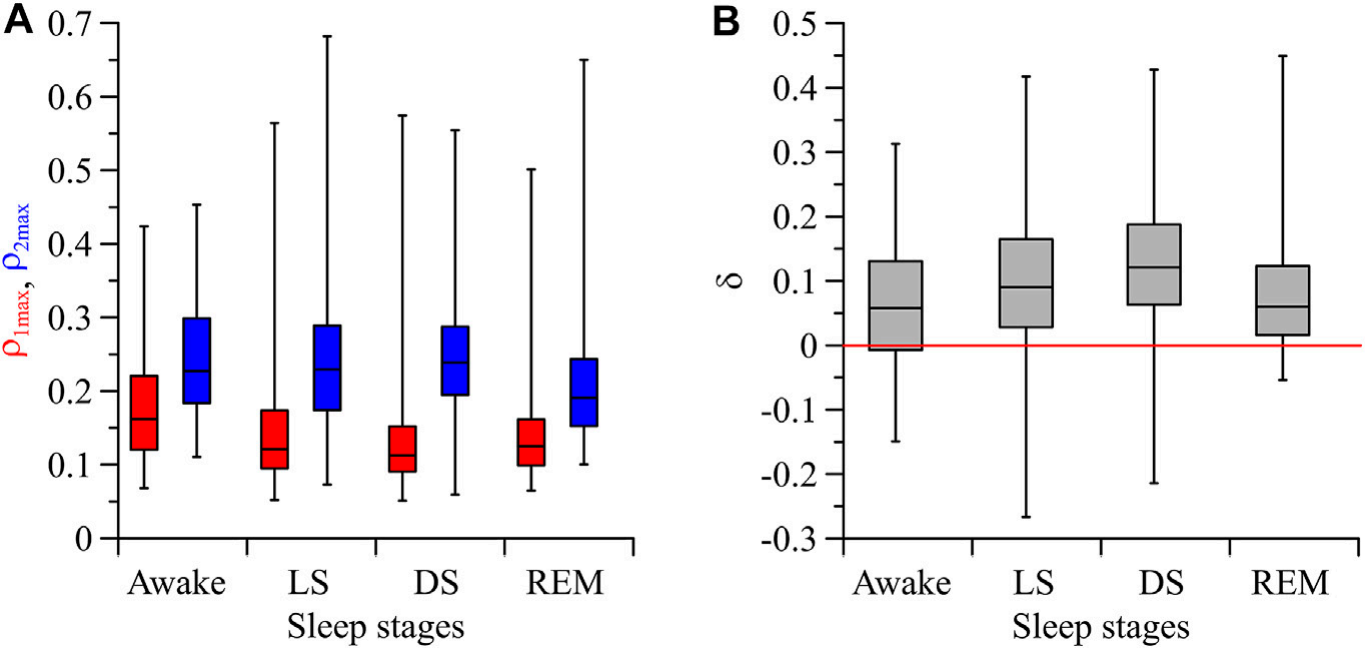
**(A)** Box-and-whisker diagrams for the values of $\rho_{1\max}$ (red) and $\rho_{2\max}$ (blue) in the awake state, LS, DS, and REM sleep for all subjects. **(B)** Box-and-whisker diagrams for the values of *δ* in the awake state, LS, DS, and REM sleep. Both in **(A)** and **(B)**, the box boundaries are the first and third quartiles, the horizontal line is the median, and the whiskers are the minimum and maximum values.

Figure 3A shows the statistically significant (*p* = 0.05) indices $\rho_{1\max}$ and $\rho_{2\max}$ for the subjects in the awake state, LS, DS, and REM sleep. In LS and DS, the difference between the indices $\rho_{2\max}$ and $\rho_{1\max}$ is greater than in the awake state and REM sleep. During LS and DS, the index $\rho_{1\max}$ takes the vales 0.16 ± 0.01 and 0.14 ± 0.01, respectively, while the index $\rho_{2\max}$ takes the values 0.25 ± 0.01 and 0.25 ± 0.01, respectively. The statistical significance of difference in indices $\rho_{2\max}$ and $\rho_{1\max}$ in the sleep stages LS and DS is confirmed by the Mann-Whitney U-test (*p* < 0.001 for both stages). The index *δ* for the subjects in the awake state, LS, DS, and REM sleep is presented in Figure 3B. During LS and DS, the index *δ* takes the values 0.09 ± 0.01 and 0.12 ± 0.01, respectively. These *δ* values are greater than in the awake state and REM sleep. Thus, it follows from Figure 3 that the asymmetry of coupling between the respiration and the process of parasympathetic control of the heart rate is most pronounced in DS.

**FIGURE 4:**
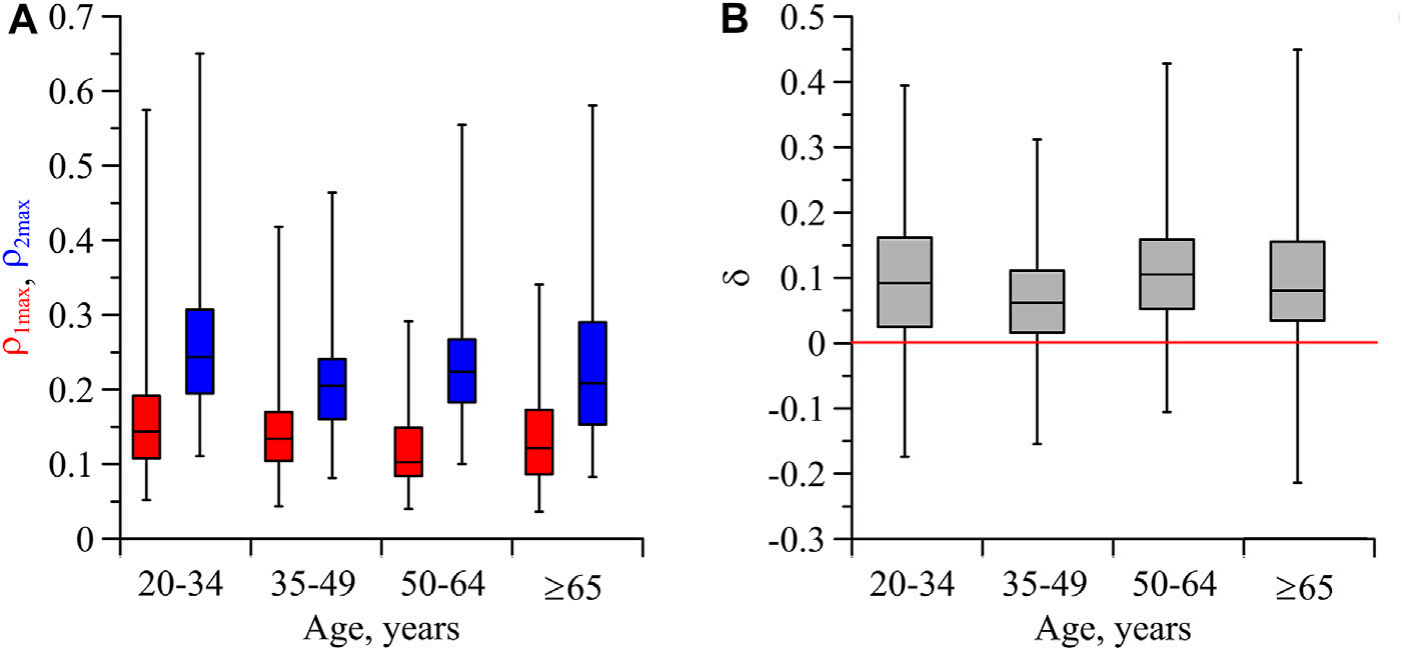
**(A)** Box-and-whisker diagrams for the values of $\rho_{1\max}$ (red) and $\rho_{2\max}$ (blue) for the subjects of different age. **(B)** Box-and-whisker diagrams for the values of *δ* for the subjects of different age. Both in **(A)** and **(B)**, the box boundaries are the first and third quartiles, the horizontal line is the median, and the whiskers are the minimum and maximum values.

Figure 4A shows the indices of directional coupling $\rho_{1\max}$ and $\rho_{2\max}$ in different age groups without taking into account the stages of sleep or wakefulness. As can be seen from this figure, both $\rho_{1\max}$ and $\rho_{2\max}$ take close values at different ages of the subjects. In the group of subjects aged 20–34 years, $\rho_{1\max}$ and $\rho_{2\max}$ were 0.16 ± 0.01 and 0.26 ± 0.01, respectively. In the group of subjects in ages 65 and older, $\rho_{1\max}$ and $\rho_{2\max}$ were 0.14 ± 0.01 and 0.23 ± 0.02, respectively. The index *δ* also turned out to be non-sensitive to the age of the subjects (Figure 4B).

## Discussion

In the present study, we analyzed the directional couplings between the respiration and the process of parasympathetic control of the heart rate in healthy subjects. It is known that parasympathetic fibers innervate the smooth muscle tone of the respiratory tract, providing regulation of microvasculature in the respiratory tract and realizing a direct directional coupling from parasympathetic regulation to the respiration. We understand that the respiratory tract is complex and involves several different muscle groups along upper airways, and for respiratory work such as the diaphragm. At the same time, feedback loops from the pulmonary stretch receptors and arterial baroreceptors act through the nucleus tractus solitarii on the Bötzinger complex located in the pontomedullary region of the pons, which provides regulation of the cardiovagal parasympathetic outflow by the respiratory pattern generator (Guyenet, 2014). Besides, the heart rate also responds to intrathoracic pressure changes caused by the respiration cycle (Berntson et al., 1993).

Taking into account the complex structure of interactions between the elements involved in the cardiorespiratory interaction, the obtained results can be interpreted as the presence of a dominant influence of respiration on the set of factors that form oscillations in the HF range of RR-intervals (in particular, vagal activity). The influence in the opposite direction is less pronounced. Moreover, it turns out that the degree of asymmetry in these directional couplings depends on the subject’s psychophysical state, which changes in different stages of sleep.

The obtained results are consistent with the results of studies, in which the dominant direction of coupling from respiration to the main heart rhythm was observed for healthy subjects of different ages (Faes et al., 2004; Faes and Nollo, 2010; Porta et al., 2012; Iatsenko et al., 2013) and it was found to be independent of subject gender (Mrowka et al., 2003; Bartsch et al., 2007; Bahraminasab et al., 2008; Shiogai et al., 2010) or sleep stage (Mrowka et al., 2003).

Moreover, the asymmetry in the coupling is more pronounced in LS and DS compared to the awake state and REM-sleep. This indicates the influence of the sympatho-vagal balance on the direction of coupling between the studied processes. The mean value of the index $\rho_{1\max}$ in LS decreases and reaches the lowest value in DS, which leads to an increase in the asymmetry in the coupling (Figure 3).

At the same time, it was shown that under certain conditions (e.g., anesthesia) the dominant direction of coupling could be from the heart to respiration (Galletly and Larsen, 1999). Although this work considered a different frequency range, associated mainly with the main frequency of the heart rate, such conclusions indicate the relationship between the dominant direction of the cardiorespiratory interaction and the psychophysical state of the subject.

To calculate the indices $\rho_{1\max}$ and $\rho_{2\max}$ , we used the method based on the phase dynamics modeling, which allows one to reveal causal relationships between the oscillatory processes in contrast to the methods for estimating the linear relationship between the signals (Anrep et al., 1936; Angelone and Coulter, 1964; Song and Lehrer, 2003; Faes et al., 2004; Milde et al., 2011; Porta et al., 2012) including the time series of phases of the cardiac and respiratory systems (Schäfer et al., 1998; Schäfer et al., 1999; Prokhorov et al., 2005b; Bartsch et al., 2007; Musizza et al., 2007; Karavaev et al., 2009). Since the signals of the studied systems demonstrate complex non-stationary dynamics (Lombardi, 2000; Karavaev et al., 2019), the method of nonlinear analysis of phase dynamics employed in our study has advantages over linear methods analyzing only amplitude dynamics to reveal the couplings.

We detected the bidirectional coupling between the respiration and parasympathetic control of the heart rate in healthy subjects at different ages both during sleep and wakefulness. This result is consistent with the results of the studies (Galletly and Larsen, 1999; Iatsenko et al., 2013), which reported the presence of bidirectional interaction between the main heart rhythm and respiration. However, our result contradicts the hypothesis that the coupling between the respiratory and cardiovascular systems is unidirectional, i.e., the respiratory rhythm affects the heart rate through stimulation of the vagus nerve (Guyton, 1991) and direct mechanical action on the sinus node (Bernardi et al., 1990; Faes and Nollo, 2010), while the influence in the opposite direction is absent. However, the influence of the cardiovascular system on the respiratory system was reported in newborns (Rosenblum et al., 2002) and in subjects with apnea (Schreiber, 2000; Bhattacharya et al., 2003; Verdes, 2005).

In our study, we found that the direction of coupling from respiration to the process of parasympathetic control of the heart rate is dominant in all age groups of subjects. Moreover, the values of the directional coupling indices in different age groups take close values. It should be noted that a decrease in cardiorespiratory phase synchronization has been found in elderly subjects (Bliwise, 1993; Shiogai et al., 2010; Bartsch et al., 2012) and a decrease in coherence between the respiration and parasympathetic control of the heart rate with aging has been reported (Ponomarenko et al., 2021). Our results indicate that the mentioned effects of decrease in coherence and synchronization of the cardiac and respiratory systems during aging occur for reasons unrelated to the values of indices of directional coupling between the respiration and parasympathetic control of the heart rate.

To the best of our knowledge, there are no special studies which indicate the presence of time delays in couplings between the respiration and HF oscillations in RR-intervals. However, there is a number of indirect evidence of the possible presence of such delays. In particular, in experiments with direct stimulation of the sympathetic and parasympathetic nerves innervating the heart, there was a delay of tens and hundreds of milliseconds in the response of the cardiovascular system to such stimulation (Warner and Russell, 1969; Somsen et al., 1985; Fagius et al., 1987; Salata and Zipes, 1991; Berntson et al., 1993).

We used the surrogate data analysis to test the hypothesis that the indices of directional coupling calculated from experimental data are significantly different from zero. Surrogate data were generated by random choice of pairs of signals from different subjects, which were not coupled by default, but had similar characteristics. Note that for each state and each direction of coupling, its own 95%-threshold was formed, above which the indices of directional coupling were considered significant.

Using the Mann-Whitney U-test, we tested the null-hypothesis about the equality of $\rho_{1\max}$ and $\rho_{2\max}$ . The test statistically significantly disproved this null hypothesis. However, it should be taken into account that the formulation of the null hypothesis when analyzing the signals from complex systems is a difficult task. For the correct interpretation of the results obtained using the quantitative assessment methods, it is necessary to take into account a large number of factors, including the information about the features of the dynamics of the systems under study. In particular, it would be useful to take into account the influence of nonstationarity of the analyzed signals and the properties of phase noises. However, the complexity of the physiological systems under study limits our possibilities. We have to confine ourselves to the assumption of an insignificant effect of the phase variance on the values of the indices $\rho_{1\max}$ and $\rho_{2\max}$ . A more detailed study of this issue requires investigation of numerical models, the signals of which reflect the statistical and dynamical properties of the phases of analyzed experimental signals.

## Conclusion

We have revealed the presence of bidirectional coupling between the respiration and the process of parasympathetic control of the heart rate during wakefulness and different stages of sleep in healthy subjects. It is found that in all age groups of subjects, the direction of coupling from respiration to the process of parasympathetic control of the heart rate is dominant. The asymmetry in coupling between the considered processes is most pronounced during deep sleep. This supports the fact that deep sleep is most important for physical restoration with energy saving behavior of physiological systems.

The obtained results provide useful additional information about the features of the cardiorespiratory interaction associated with the modulation of regulatory processes by the higher nervous activity. Furthermore, the considered indices of directional coupling can be useful in sleep studies as an additional tool for classifying sleep stages without registration of electroencephalograms.

## Data Availability

The data analyzed in this study is subject to the following licenses/restrictions: these are data which belong to medical faculties and are not publicly available. Requests to access these datasets should be directed to TP, thomas.penzel@charite.de.
